# Characteristics of DNA macro-alterations in breast cancer with liver metastasis before treatment

**DOI:** 10.1186/s12864-023-09497-w

**Published:** 2023-07-11

**Authors:** Yu Fan, Linglin Zou, Xiaorong Zhong, Zhu Wang, Yu Wang, Chuanxu Luo, Hong Zheng, Yanping Wang

**Affiliations:** Breast Center and Multi-Omics Laboratory of Breast Diseases, West China Hospital, Sichuan University, 5 Gongxing Street, Wuhou District, Chengdu, 610041 China

**Keywords:** Breast Cancer, Liver Metastasis, Macro-alteration, Whole-genome Doubling, Complex structural variation

## Abstract

**Background:**

Whole-genome doubling (WGD) has been observed in 30% of cancers, followed by a highly complex rearranged karyotype unfavourable to breast cancer's outcome. However, the macro-alterations that characterise liver metastasis in breast cancer(BC) are poorly understood. Here, we conducted a whole-genome sequencing analysis of liver metastases to explore the status and the time frame model of these macro-alterations in pre-treatment patients with metastatic breast cancer.

**Results:**

Whole-genome sequencing was conducted in 11 paired primary tumours, lymph node metastasis, and liver metastasis fresh samples from four patients with late-stage breast cancer. We also chose five postoperative frozen specimens from patients with early-stage breast cancer before any treatment as control. Surprisingly, all four liver metastasis samples were classified as WGD + . However, the previous study reported that WGD happened in 30% of cancers and 2/5 in our early-stage samples. WGD was not observed in the two separate primary tumours and one lymph node metastasis of one patient with metastatic BC, but her liver metastasis showed an early burst of bi-allelic copy number gain. The phylogenetic tree proves her 4 tumour samples were the polyclonal origin and only one WGD + clone metastasis to the liver. Another 3 metastatic BC patients’ primary tumour and lymph node metastasis experienced WGD as well as liver metastasis, and they all showed similar molecular time-frame of copy number(CN) gain across locations within the same patient. These patients’ tumours were of monoclonal origin, and WGD happened in a founding clone before metastasis, explaining that all samples share the CN-gain time frame.

After WGD, the genomes usually face instability to evolve other macro-alterations. For example, a greater quantity and variety of complex structural variations (SVs) were detected in WGD + samples. The breakpoints were enriched in the chr17: 39 Mb-40 Mb tile, which contained the *HER2* gene, resulting in the formation of tyfonas, breakage-fusion-bridge cycles, and double minutes. These complex SVs may be involved in the evolutionary mechanisms of the dramatic increase of *HER2* copy number.

**Conclusion:**

Our work revealed that the WGD + clone might be a critical evolution step for liver metastasis and favoured following complex SV of breast cancer.

**Supplementary Information:**

The online version contains supplementary material available at 10.1186/s12864-023-09497-w.

## Introduction

Metastatic breast cancer (BC) may be biologically different from the original primary tumour because cancer cells continue to evolve stochastically or in response to treatment/microenvironment, leading to treatment failure and death [1]. Evidence indicates that hormone receptors and human epidermal growth factor receptor 2 (HER2) status are discordant between primary and metastatic tumours in 20%-25% of patients [2]. Therefore, it is necessary to understand the genomic profile that depicts metastasis and evolution. Most molecular studies of metastatic BC have been based on single-nucleotide variants (SNVs) and fusion genes by single rearrangements [3–5]. Nevertheless, macro-alterations that significantly impact the malignant phenotype and prognosis [6–8] are poorly understood.

Macro-alterations include whole-genome doubling (WGD), somatic copy number alterations (CNAs), complex structural variations (SV), and other similar fatal events to survival [9] that may occur instantaneously during a particular developmental stage in cancer cells. In most cases, these events are presumed to be harmful, and only in some rare cases does it result in an increase in cellular fitness and the generation of viable "hopeful monsters" [10]. Doubling of a complete set of diploid chromosomes has been proposed as a very early event in tumour evolution [11, 12], resulting in tetraploidy, and cancer cells experience missegregation or extensive loss of heterozygosity (LOH) events to evolve a more stable aneuploidy genome [13]. Complex SV refers to multiple (> 2) DNA junctions in distinct topologies within the reference genome that yield one or more copies of complex rearranged alleles [14]. Tyfonas represent an extreme case that harbours significantly elevated junctions [15]. Together with others, these macro-alterations represent an alternative evolutionary pattern different from the Darwinian selection. However, one concern is whether these macro-alterations appear before or after metastasis. Treatment such as chemo-radiation therapy makes it more complicated because there are 2 possibilities. The first is that the treatment selects resistant sub-clones that have already evolved macro-alterations [16, 17]. Another is that these macro-alterations occur following exposure to therapeutics, which act as stressors to promote adaptive, genome-based evolution [18]. Hence, this study used whole-genome sequencing (WGS) data to evaluate these macro-alterations of BC in simultaneous paired fresh primary tumours, liver metastases, and lymph node metastases samples from four patients with late-stage BC. They were all treatment-naive.

We detected WGD events in all four liver metastases. In one of the late-stage patients, WGD occurred only in liver metastases with punctuated copy number (CN) gain at a very early molecular stage of cell development. In the other three patients, WGD occurred across all specimens that showed a similar limited or wide distribution of the CN-gain time frame. In addition, complex SVs breakpoints were mainly present in WGD + samples. Our work implied macro-alterations in our samples were not the result of treatment stress or the liver's microenvironment. They might happen stochastically in the early molecular time of cell development.

## Results

### Patient characteristics

Four patients with liver metastatic BC (MBC) at diagnosis and five with early BC (EBC) were enrolled. A CT-guided core needle biopsy was used to acquire primary tumours (pri), lymph node metastasis (ln) (MBC1 and MBC2), and liver metastasis (liv) samples. MBC1 had two separate primary tumours, and we punctured both. MBC3 had more than two primary tumours; we only punctured the largest one because the others were too small to puncture. Five postoperative specimens of early-stage primary tumours stored at -80 °C were obtained from the West China Hospital Breast Cancer Biobank [19]. All samples were treatment-naive. The molecular subtypes, treatments, and outcomes are summarised in Supplemental Table 1. Hormone receptor status of primary tumours was positive in MBC2, MBC3, MBC4, EBC3, EBC6, EBC7, and EBC8 and negative in MBC1 and EBC1. HER2 status of primary tumours was positive in MBC1, MBC2, MBC4, EBC3, EBC6, EBC7, and EBC8 and unknown in MBC3 and EBC1. Overall survival ranged from 125–152 months in EBCs and 11.8–70.1 months in MBCs. Progression-free survival ranged from 0–23.37 months in MBCs.

### Mutational loads and signature

**Fig. 1:**
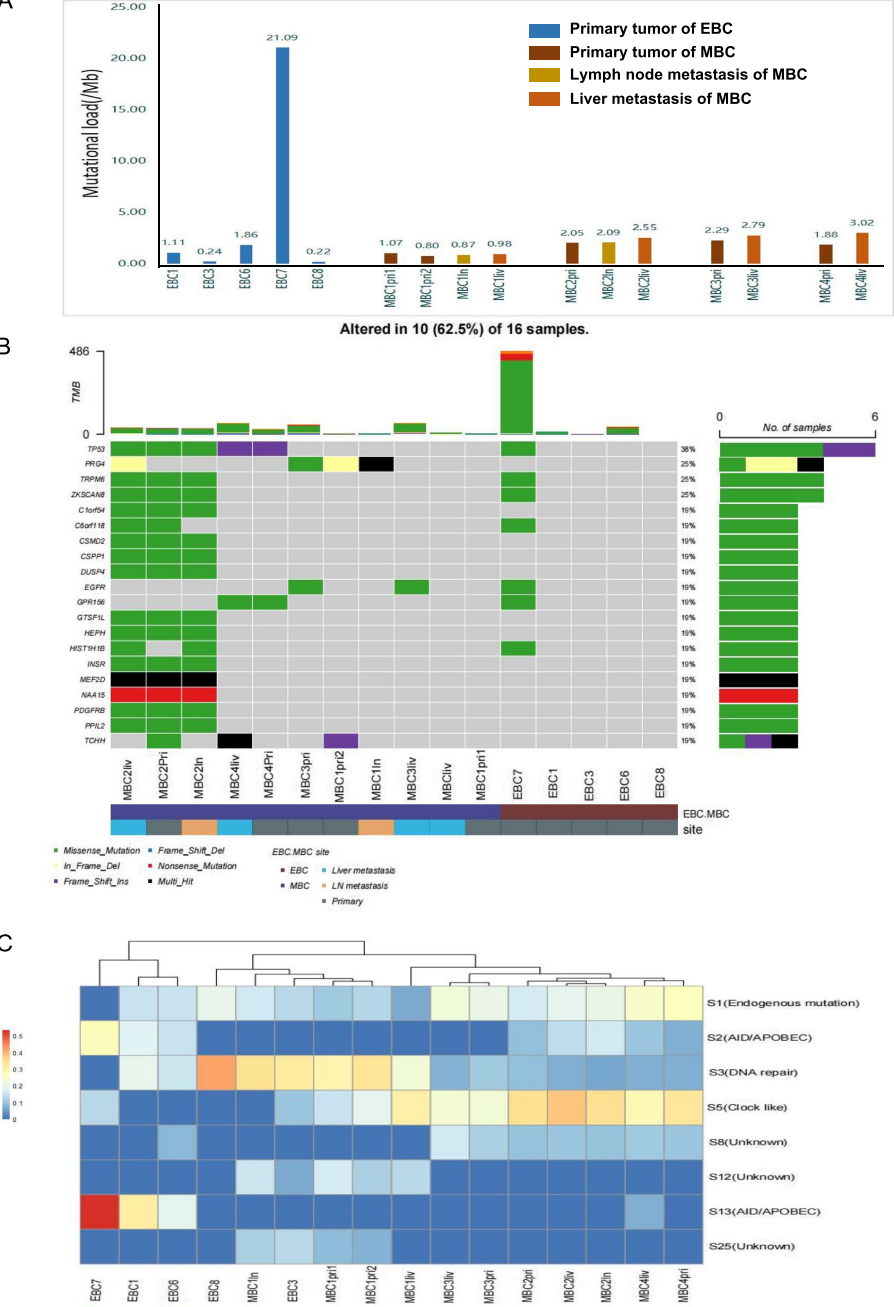
Mutational loads and signature of early- and late-stage breast cancer. **A** Mutation loads per megabase for each sample. pri, primary tumour sample; ln, lymph node metastasis; liv, liver metastasis. **B** Top 20 mutated genes in early or late disease. **C** Mutational signatures in early and late diseases. Blue indicates that the normalised inferred weight of the indicated signature is 0, and red indicates 0.5. The patients were clustered according to the weight of the mutational signatures

All tumour samples underwent WGS (coverage range 24–37'). Blood or normal breast tissues were used as normal controls. BC has a moderate mutational load, which has been reported to be close to one mutation/Mb [20]. In our EBC samples, the mutational loads ranged from 0.22 (EBC8) to 21.09 (EBC7) mutations/Mb. In MBCs, the mutational loads ranged from 0.8 (MBC1pri2) to 3.02 (MBC4liv) mutations/Mb (Fig. 1A, Supplemental Table 2). The most commonly mutated cancer driver genes were *TP53* (patient number(P) = 3,sample number(*N*) = 6), *EGFR* (*P* = 2,*N* = 3) and *RECQL4*(*P* = 2,*N* = 2). Other driver gene mutations did not occur in more than two patients (Fig. 1B, Supplemental Table 3). No cancer driver gene mutations were detected in the 4 samples of MBC1. For MBC2-MBC4, 43.13%-49.22% SNVs of the primary tumour was found to be overlapped with liver metastasis SNV. Nevertheless, only 1.38% SNVs of MBC1pri1 and 3.39% SNVs of MBCpri2 overlapped with liver metastasis SNV(Supplemental Fig. 1).

To understand whether there were differences in the mutational signature between primary tumours and liver metastases, we estimated the mutational signature of each sample based on COSMIC v2 (Fig. 1C, Supplemental Table 4). MBC1pri1, MBC1pri2, MBC1ln, EBC3, and EBC8, which had the lowest mutational load of all samples, were dominated by signature 3 (range 0.29–0.42). All samples of MBC2, MBC3, and MBC4 were dominated by signature 1 (range 0.17–0.27) and signature 5 (range 0.23–0.38). MBC1liv had both signature 3 (0.23), similar to other samples of MBC1, and signature 5 (0.31), similar to the other samples of late-stage patients. The other three patients with early-stage BC (EBC1, EBC6, and EBC7) were dominated by signatures 2 (range 0.15–0.27) and 13 (range 0.20–0.53). Signature 3 is associated with the failure of DNA double-strand break repair by homologous recombination, and signature 5 was previously associated with age (clock-like) in several different cancers [21]. Signature 2 + 13 shows activation of AID/APOBEC cytidine deaminases [20]. EBC7 had our samples' highest mutational load of 21.09 mutations/Mb. It also showed the highest signature of AID/APOBEC cytidine deaminases, which were reported as the most common mutational processes among hypermutated breast cancer [22].

Interestingly, even though the SNVs/indels of liver and lymph metastases differed almost entirely from the two primary tumours in MBC1, they still showed some similarity in signature, suggesting the tumour samples from MBC1 were from the different clones with the same pathogenic factor.

### WGD and LOH

**Fig. 2:**
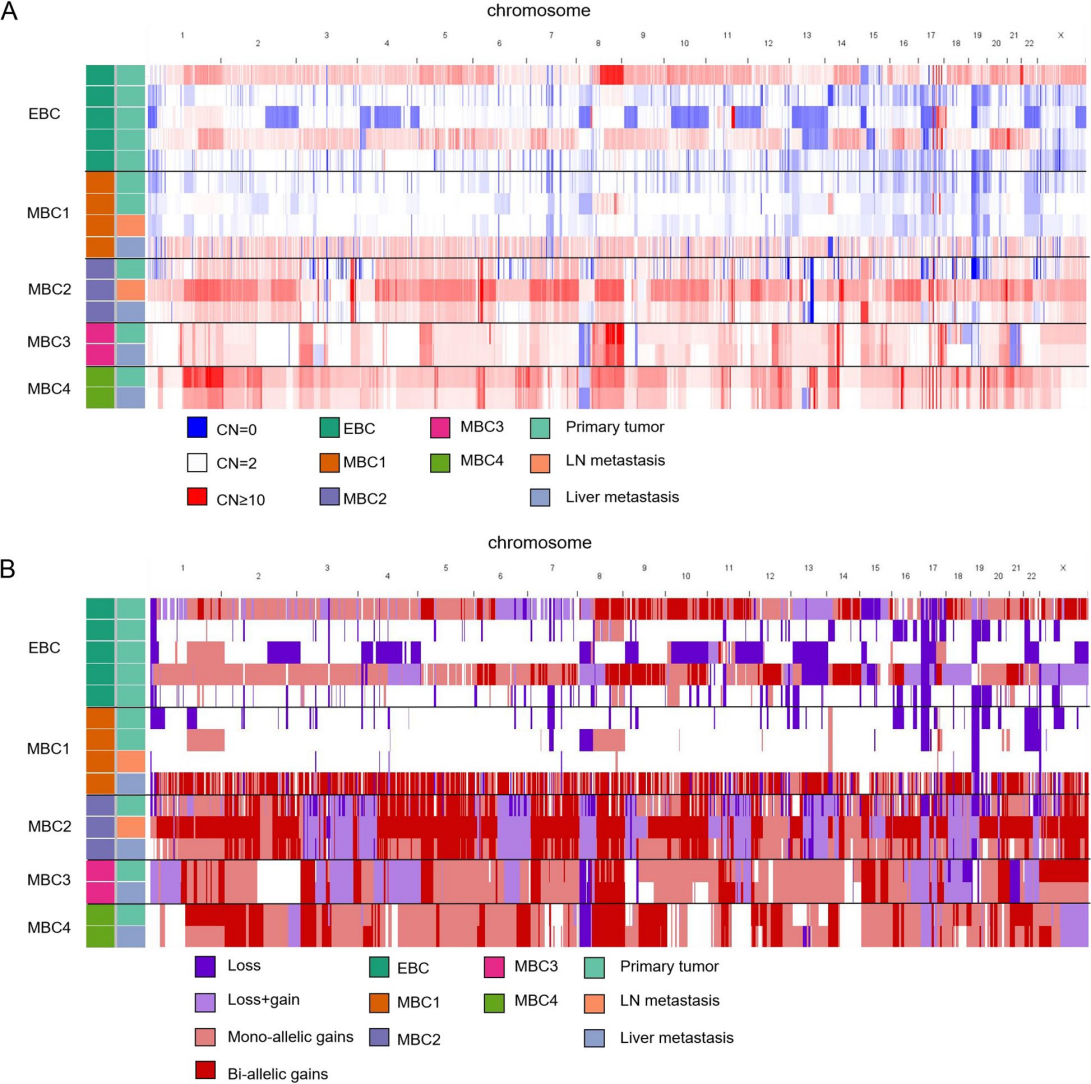
Patients with late-stage breast cancer showed similar copy number alterations (CNAs) and patterns of allele-specific gain and loss across different tissues except for the polyclonal tumour. **A** Segmental CNAs in each sample. The same colour represents regions with a total copy number > 10. **B** Left segmental allele-specific gain/loss in each sample. Dark red, bi-allelic gain; light red, mono-allelic gain; light purple, one allele gain and another allele loss; dark purple, one allele loss and another allele normal or loss

In MBC1liv, 52.29% of the genome was affected by the bi-allele gain (allele CN N: N, N ≥ 2), 24.64% was affected by the mono-allele gain (N:1), 2.73% was affected by gain + loss (N:0), and 9.22% was affected by loss (1:0). MBC1liv was classified as WGD + , as 50% or more of the autosomal tumour genome had a somatic major CN of two or more [6]. However, the other three samples from MBC1 were WGD-, with a gain-affected genome range of 0.72%-9.81%. Again, this implied that the tumours from MBC1 were of poly-clone origin, and only the clone metastases to the liver underwent WGD. In contrast, in MBC2, MBC3, and MBC4, allele-specific CNAs typically involved the same alleles within the same patients, suggesting a common seeding of these samples (Fig. 2A, Supplemental Table 5). All samples from MBC2, MBC3, and MBC4 were classified as having undergone WGD (gain-affected genome range: 78.35%–99.09%) (Fig. 2B, Supplemental Table 5). 2/5 EBCs had WGD events(Supplemental Table 5). The ploidy of WGD + samples ranged from 2.92–5, and that of WGD- samples ranged from 1.78–2.04. All TP53-mutated samples from three patients were WGD + . However, four WGD + samples (EBC1, MBC1liv, MBC3pri, and MBC3liv) did not harbour *TP53* mutations, suggesting that *TP53* dysfunction is not obligatory for WGD [6].

Tumour cells were reported to tolerate many large-scale losses after WGD to evolve a more stable sub-tetraploid tumour genome [6], and WGD buffers the deleterious impact of somatic mutations and somatic CNAs in regions of LOH [23]. Allele loss affected 8.17%-35.98% of the genome of WGD + samples and 1.63%-30.26% of the genome of WGD- samples. In other words, our limited samples did not observe a correlation between WGD and LOH. Recurrent loss of 8p23.3-8p12, 17p, and 17q21.2-17q21.31 resulted in the LOH of *TP53* in 14/16 samples and *BRCA1* in 13/16 samples.

### Sub-clonal composition and evolution

**Fig. 3:**
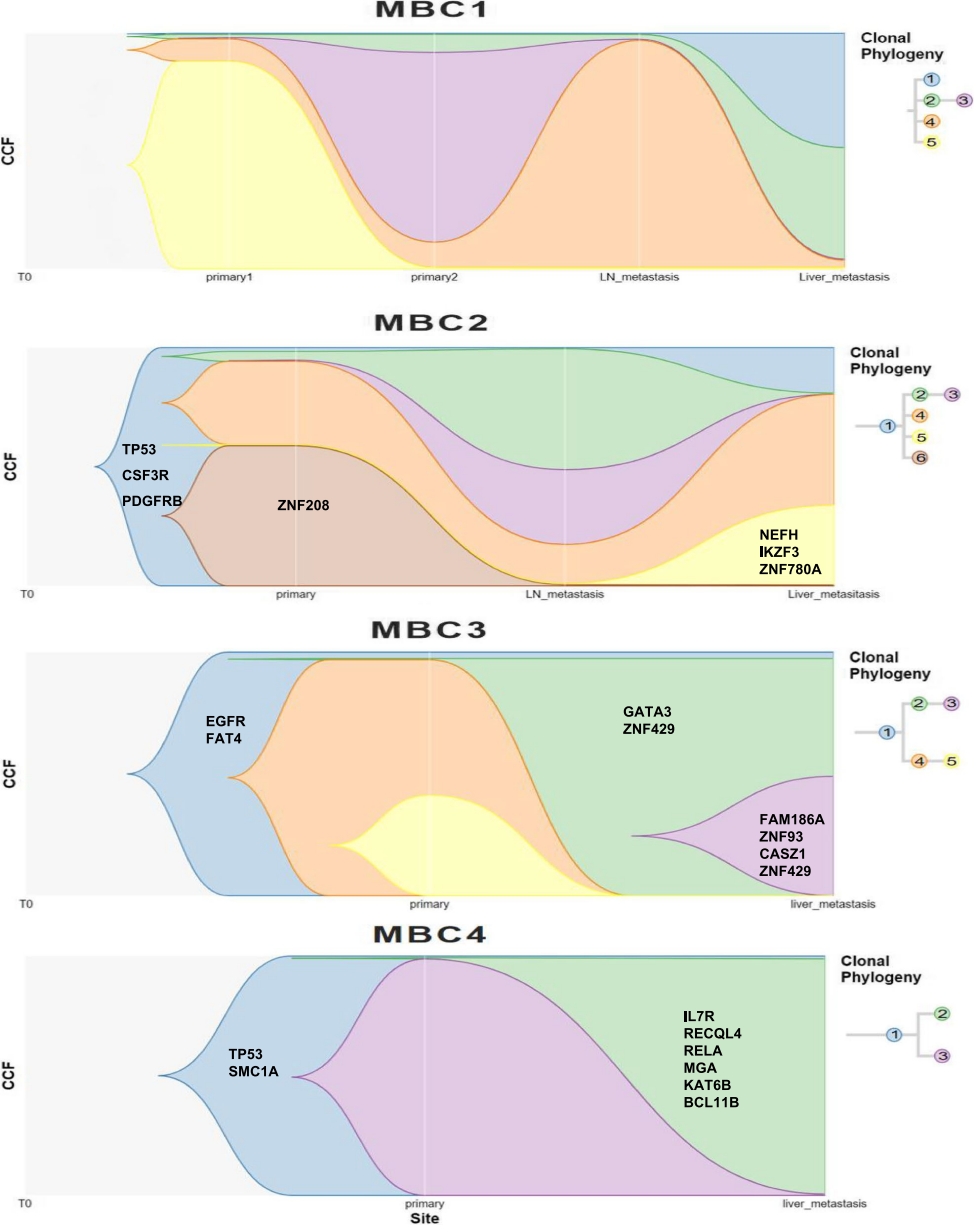
Cancer cell fraction and phylogenetic trees revealed the monoclonal and polyclonal origin of the four patients with late-stage breast cancer. Copy number and single nucleotide variation/indel-based phylogenetic trees of patients with liver metastasis. The cancer cell fractions (vertical axis) are plotted across regions (primary, lymph node, and liver metastases) for each clone (colours), and the evolutionary relationships between clones are shown by the right phylogenetic tree and timescape layout

We used phylowgs to infer the sub-clones and build a phylogenetic tree from SNVs and CNAs. It is widely accepted that tumours are monoclonal in origin, arising from a series of mutations in a single cell and its descendants [24] MBC2, MBC3, and MBC4 originated from a founding clone with cancer driver gene mutations established in the primary tumour seeding all other metastases (Fig. 3). The founding clone then diverged into 2–4 lineages predominantly present in primary tumours, lymph node metastasis, or liver metastasis. Some private clones then evolved from these lineages, such as clone 3, which evolved from clone 2, and were both present in the liver metastasis of MBC3. However, polyclonal lesions, characterised by distinct cell subpopulations expanding within separate domains of the growing tumour, were also reportedly present in various tumours [25]. Clones 1, 2, 4, and 5 of MBC1 were presumed to have polyclonal origins. Clone 1, characterised by vast CNAs, was predominantly present in liver metastases (Supplemental Table 6).

In addition, MBC1 primary tumour was clinically diagnosed as a HER2-enriched subgroup using immunohistochemistry (IHC) and fluorescent in-situ hybridisation (FISH). The two primary tumours' *HER2* CNs calculated by WGS were 5 and 18, in line with clinical diagnosis. However, the *HER2* CNs in the lymph and liver metastases were 3 and 3, respectively(Supplemental Table 7), suggesting they lost the HER2 amplification. Progression-free survival was only 3.5 months after trastuzumab treatment for brain metastasis, and 1 year later, liver metastasis had progressed. Another patient, MBC4, who was diagnosed as HER2 positive of the primary tumour and *HER2* CN were 49(pri) and 51(liv) calculated by WGS data, had a 23.37 months PFS(supplemental Table 1) after treatment with trastuzumab. The dominance of clones without *HER2* amplification of MBC1 may be one reason for trastuzumab resistance. In MBC2, MBC3, and MBC4, the *HER2* CN was constant between primary and metastatic tumours. The heterogeneity of metastatic BC may underlie its poor responsiveness to therapy and explain why biomarkers of therapy responsiveness measured exclusively in primary tumours provide a restricted view of the biological properties of metastatic BC.

### Complex SV

**Fig. 4:**
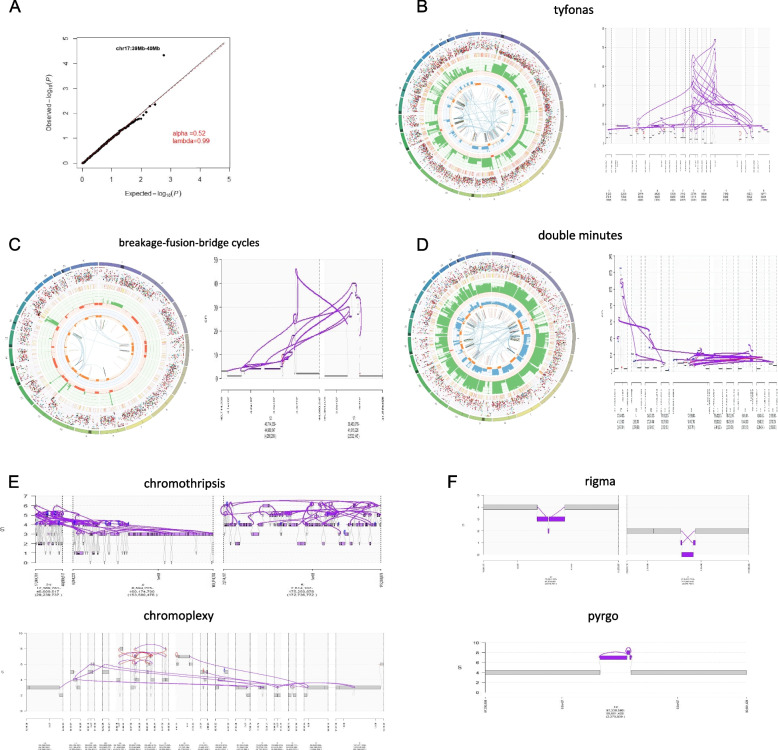
The breakpoint enriched in the tile of chr17: 39 Mb–40 Mb and WGD + samples harboured complex SVs. **A** Quantile–quantile plots showing *p*-values for breakpoint enrichment tiles with intervals of 1 Mb across the 16 samples. **B**-**D** Three types of complex SVs caused dramatic copy number elevations, including tyfonas in MBC4liv(B), breakage-fusion-bridge cycles in EBC6(C), and double minutes in MBC2ln(D). Left, the circle from outer to inner shows chromosomes, single nucleotide variations/indels, all observed tumour purity-adjusted copy number changes, minor allele copy numbers’ across the chromosome, and the observed structural variants within or between the chromosomes, respectively. Right, JaBbA graph of DNA segments and bonds involved in the three types of complex SVs. **E**–**F** JaBbA graph of the DNA segments and bonds involved in four types of complex SVs that cause low DNA copy number changes. Chromothripsis in EBC7 and chromoplexy in MBC4liv(E) occurred in a wide range of chromosomes. Rigma in MBC3liv and pyrgo in MBC3 liv(F) had a relatively narrow range and a single chromosome. WGD, whole-genome doubling; SV, structural variation

Another DNA macro-alteration is complex SV, which was reported to be accelerated in WGD + cells [26]. In our study, All complex SV types, including tyfonas, breakage-fusion-bridge cycles (BFBs), chromoplexy, chromothripsis, double minutes (DMs), pyrgo, and rigma, were detected in 9/10 WGD + and 3/6 WGD- samples (Table 1). In the three WGD- samples, only 1–2 complex SVs were detected. In nine WGD + samples, more than five complex SVs or tyfonas were detected, which were highly complicated with high junction-CN junctions and fold-back inversions [15]. The breakpoints of the complex SVs enriched in the chr17: 39 Mb-40 Mb tile (*p* = 5.80E-06, false discovery rate = 0.017) (Fig. 4A, Supplemental Table 8), which recurrently involved the copy number change of *HER2*.

**Table 1:** The WGD status and the complex and simple structural variants number in each sample

samples	WGD	complex							simple			
		tyfonas	bfb	dm	chromoplexy	chromothripsis	rigma	pyrgo	DEL-like	DUP-like	INV-like	TRA-like
EBC1T	+	1	0	0	0	0	0	0	17	14	22	113
EBC3T	-	0	0	0	0	0	0	0	0	1	3	0
EBC6T	-	0	1	0	1	0	0	0	9	6	24	26
EBC7T	+	0	1	0	6	2	1	0	46	37	63	58
EBC8T	-	0	0	0	0	0	0	0	1	0	1	8
MBC1Pri1	-	0	0	0	0	0	0	0	4	6	0	3
MBC1Pri2	-	0	1	0	0	0	0	0	23	21	33	3
MBC1ln	-	0	0	0	0	1	0	0	2	6	6	0
MBC1liv	+	0	0	0	0	0	0	0	4	2	36	28
MBC2pri	+	0	0	1	4	0	0	0	32	26	74	112
MBC2ln	+	0	1	4	5	0	0	0	31	25	84	130
MBC2liv	+	0	1	1	5	0	0	0	31	25	77	108
MBC3pri	+	0	0	1	6	0	1	0	142	141	279	96
MBC3liv	+	0	0	0	3	0	2	1	58	49	91	63
MBC4pri	+	1	1	1	5	1	0	0	44	35	77	91
MBC4liv	+	1	1	0	4	1	0	0	45	33	89	95

The amplitude of CN was relatively flat in the samples without complex SVs, whereas steep amplification peaks were observed in the samples with complex SVs. In EBC1 (*HER2* CN = 29), MBC4pri (*HER2* CN = 49), and MBC4liv (*HER2* CN = 51), tyfonas were implicated in *HER2* amplification (Fig. 4B). BFBs and DMs were also associated with *HER2* amplification (Fig. 4C-D). Hence, the HER2-enriched samples diagnosed using IHC (3 +), FISH ( +), or CN by WGS (≥ 6) could be divided into subgroups with or without complex SVs. The HER2-enriched samples with complex SVs around *HER2* showed a higher CN of *HER2* (range 18–129) compared with the HER2-enriched samples without complex SVs (range 4–8). Chromoplexy was the most prevalent event type (events = 39, patients *n* = 9) (Table 1), which affected the genome with low DNA CN changes(Fig. 4E). Chromothripsis (events = 5, *n* = 4)(Fig. 4E), rigma (events = 4, *n* = 3), and pyrgo (events = 1, *n* = 1) were less frequent (Fig. 4F) in our samples.

We verified this result in a published database [15]. There were 257 breast cancer samples in the database, of which 5 were metastatic breast cancer samples. 3 samples underwent WGD (supplementary Fig. 2), and 2/3 of the WGD sample showed a loss of function of TP53. A total of 37 complex SV were detected in these 5 samples, and similar to our results, 36/37 complex SV happened in the 3 WGD samples (Supplemental Fig. 2, Supplemental Table 9).

### Timing patterns of CN-gain

**Fig. 5:**
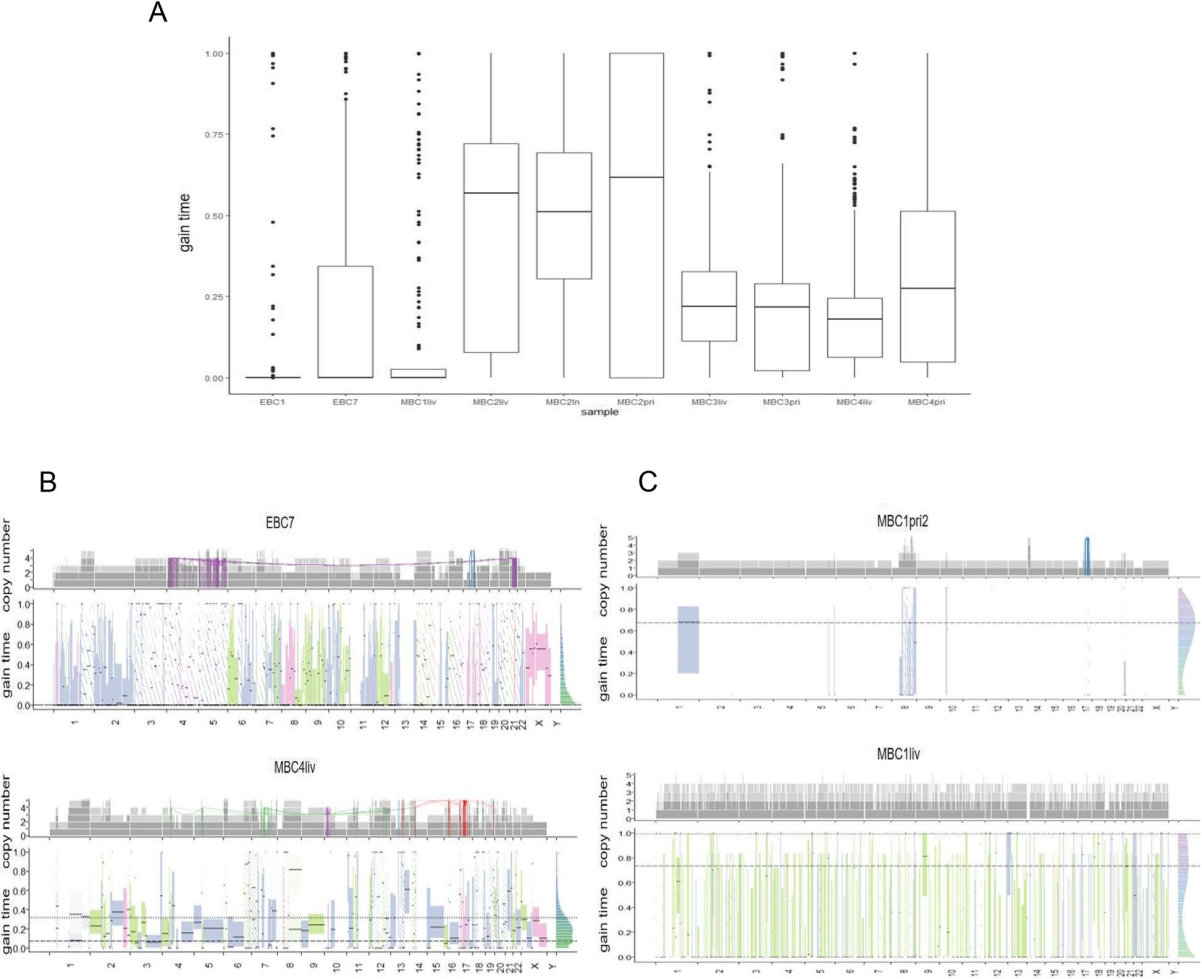
Different patterns of copy number gain time of WGD + samples. **A** Boxplot showing the distributions of molecular time of copy number gain. **B** Segmental molecular time of copy number gain in the WGD + samples of EBC7 and MBC4liv. **C** Segmental molecular time of copy number gain in the MBC1pri2 and MBC1liv. The upper plot shows the copy number of the stacked bar plots. Dark grey indicates a major allele, and light grey indicates a minor allele. Red links, tyfonas; blue, breakage-fusion-bridge cycles; purple, chromothripsis; green, chromoplexy; orange, double minutes. The bottom plot shows the estimated mutation times for the primary and secondary gain (shaded). Boxes denote 95% confidence intervals. Blue = mono-allelic gain (*n* = 1), pink = copy number-loss of heterozygosity/gain + loss (*n* = 0), and green = bi-allelic gain (*n* = 2). WGD, whole-genome doubling

We estimated the molecular time of CN-gain using the R package 'mutationtimeR.' In WGD + samples of EBC1 (median gain time 2.84E-21, IQR 5.77E-33 to 1.55E-10), EBC7 (median 2.51E-12, IQR 1.52E-57 to 0.34), MBC1liv (median 6.60E-36, IQR 3.85E-65 to 0.025), MBC3pri (median 0.22, IQR 0.022 to 0.29), MBC3liv (median 0.22, IQR 0.11 to 0.33), MBC4pri (median 0.28, IQR 0.049 to 0.51), and MBC4liv (median 0.18, IQR 0.063–0.25), a substantial fraction of CN-gain occurred early in molecular time. In contrast, CN-gain occurred at mid-molecular times in MBC2pri (median 0.62, IQR 9.99E-28 to 1), MBC2ln (median 0.51, IQR 0.3 to 0.69), and MBC2liv (median 0.57, IQR 0.078 to 0.72) (Fig. 5A). The early punctuated bursts of CN-gain detected in MBC1liv rather than in MBC1pri1, MBC1pri2, and MBC1ln supported a private WGD + clone diverging at a very early stage of tumour evolution and obtaining superior metastatic potential. Similar molecular gain times in MBC2, MBC3, and MBC4 suggested that the primary tumours and metastasis shared the founding clone, which acquired CN-gain at a fixed time before metastasis.

It was reported that WGD + cases had a higher proportion of multiple gain events (more than two) of CN segments compared with diploid cases (24.8% vs 11.4%), and 84% of secondary gain events showed a latency after the first one [27].In our samples, multiple gain-affected genomes ranged from 3.53% (MBC1liv) to 88.32% (MBC2ln) in WGD + cases and from 0 (MBC1ln) to 1.93% (EBC6) in WGD- cases (Supplemental Table 10). Fifty-six percent of bases showed a relative latency in secondary gain with available timing information (Supplemental Table 11). Interestingly, 910 of the 1058 (86.01%) complex SV breakpoints were located on the chromosome arm, which had large-scale multiple gains (≥ 3 Mb). However, only 28.09% (66/235) of the chromosome arms that underwent large-scale multiple gains had complex SVs (Fig. 5B-C, Supplemental Table 12). Therefore, cumulative local CN-gain and complex chromosome rearrangements probably arise from the same catastrophic events of chromosomal arms.

## Discussion

All four liver metastases were classified as WGD + , which implies that WGD is a prevalent event in BC metastasis, considering that WGD occurs in 19% of all types of BC, 50% of triple-negative BC [28] and 40% of our early BC samples. However, different time patterns of clonal evolution were observed according to the gain time for each CN across the samples. Generally, WGD can occur in the founding clone before diversification during a limited (MBC3, MBC4) or widely distributed time frame (MBC2). MBC1 displayed diversification of polyclonal seeding, and only the clone dominantly presented in liver metastasis underwent WGD at a very early molecular time. All TP53-mutated samples from three patients underwent WGD + , but not all WGD + samples harboured TP53 mutation, suggesting that TP53 dysfunction is not obligatory for WGD. As reported, some other genes were associated with WGD in TP53 wild-type cancer, including amplification of *CCNE1* and loss-of-function mutations in *RB1* and *BAP1* [6].

The 4 samples from MBC1 showed similar mutational signatures representing DNA double-strand break repair by homologous recombination, suggesting that the same driver factors were involved in the polyclonal tumours. Additionally, WGD has been consistently linked to metastatic potential [29]. The common consensus of evolution is that mutations and chromosomal aberrations accumulate gradually and sequentially over time [30], in line with Darwinian evolution. However, an alternative model is punctuated CN evolution, in which many chromosomal rearrangements and CN-gain are acquired in short bursts of genomic instability early in tumour evolution [31, 32]. Our multi-site biopsy data provide clinical evidence supporting the CN-gain of WGD during a limited or widely distributed time frame that may happen before diversification and any treatment or in one of the polyclones.

Nearly 90% of breakpoints that constituted complex SVs were found in the WGD + samples. These breakpoints enriched on chromosome 17q 39 Mb-40 Mb and involved special rearrangement patterns associated with high-junction CN junctions such as tyfonas, BFBs, and DMs, dramatically increasing the CN of *HER2* and nearby genes. Tyfonas have been reported to be enriched in both luminal BC and HER2 + BC [15] and are associated with *MDM2* and *CDK4* genes. However, two tyfonas events in our early- or late-stage patients affected chr17 q12-21, suggesting a novel mechanism for *HER2* amplification. Moreover, HER2-enriched patients with complex SVs nearby *HER2* showed a much higher *HER2* CN than HER2-enriched patients without complex SVs. Hence, more evidence is needed to explore the predictive efficiency of complex SVs for anti-HER2 therapy [33]. Other novel complexes SVs, such as rigma and pyrgo, were rare in the samples. Interestingly, we found that complex SVs mostly co-occurred with multiple large-scale gains of chromosome arms. There is no strict consensus in the literature; therefore, we classified large-scale secondary gain using ≥ 3 Mb as the threshold for CNAs [34]. We could not exclude the possibility that the chromosome arm had acquired this large-scale secondary CN via complex SVs. However, nearly 70% of arms with large-scale secondary gain did not have complex SVs, implying that they could be independent events. Cumulative local CN-gain and chromosome rearrangements may arise from the same catastrophic events, such as localised cycles of impaired fork progression and breakage [35].

Our results provide a comprehensive profile of macro-alterations, but more clinical samples, large postoperative specimens, and cell lines are needed for high-depth sequencing and RNA validation. To our knowledge, most primary-metastasis paired studies performed sampling at autopsy or the cancer progression time, meaning that clones were selected by therapy at that time. Our results provided a primitive tumour status and development before therapy and demonstrated that at least some tumour's macro-alteration evolved stochastically before treatment and metastasis. We also stress that the metastatic lesion does not entirely reflect the primary variation feature. Treatment should be targeted to the genetic makeup of the primary and metastatic lesions, respectively. This suggests that we need to combine primary tumour detection and non-invasive techniques for metastatic lesions, like a liquid biopsy, to comprehensively determine the biology of metastatic lesions so that patients can be treated accordingly.

## Conclusion

we used paired primary tumours and liver metastases of BC to understand the macro-alterations that may evolve before treatment or even at the very early stage of development. We also stress that primary and metastatic tumours may harbour different macro-alterations; therefore, multi-point sampling is necessary for late-stage patients before treatment, although it is not a routine practice.

## Methods

### Patients and collection of tissue specimens

From Jan 2014 to Jan 2015, four female patients diagnosed with breast invasive ductal carcinoma with incurable liver metastatic solid tumours who agreed to puncture the liver metastasis were included. Hormone receptors and HER2 statuses were confirmed using IHC and IHC/FISH, respectively. The necrotic areas of the lymph nodes and liver metastases were not beyond a quarter of the volume. CT-guided core needle biopsies of the primary tumour and lymph node and liver metastases were stored in liquid nitrogen and sent for pathological diagnoses. Postoperative specimens of the primary tumours from eight patients with EBC were stored at -80 °C. Blood or the normal breast tissue from the same patient was used as control.

This study was approved by the Clinical Test and Biomedical Ethics Committee of West China Hospital Sichuan University (2013; No. 128). All patients provided informed consent before any study procedure and complied with all relevant ethical regulations. Every late-stage patient had an outpatient or telephonic follow-up every 2 months. Clinical outcomes were evaluated using the Response Evaluation Criteria in Solid tumours v.1.1.

### DNA extraction and WGS

#### DNA extraction and WGS

DNA was isolated from 10 mg of tumour tissue according to the supplier's protocols (Qiagen, Hilden, Germany) using a QIAamp DNA Mini kit (Cat No./ID 51304). DNA was quantified by NanoDrop (ThermoFisher, Waltham, MA) and gel electrophoresis, followed by the preparation of a random fragmentation library with a DNA length of 200–500 bp. Barcoded libraries were sequenced as pools on the HiSeq2500 (Illumina, San Diego, CA) platform generating 2 × 150 read pairs using standard settings to a depth of 30.

### Somatic mutation

After trimming and quality filtering using Trim Galore (v.0.6.5, https://github.com/FelixKrueger/TrimGalore), sequencing reads were aligned to the human reference genome (hg38, https://storage.googleapis.com/genomics-public-data/resources/broad/hg38/v0/Homo_sapiens_assembly38.fasta) using the bwa mem algorithm, (v.0.7.17, https://github.com/lh3/bwa), sorted by samtools (v.1.10.2, https://github.com/samtools/samtools) [36], marked for duplication, and recalibrated using the Genome Analysis Toolkit (GATK) (v.4.1.1.0) according to the GATK best practice workflows (https://gatk.broadinstitute.org/hc/en-us/sections/360007226651-Best-Practices-Workflows).

Mutect2 called SNVs and small insertions/deletions (indels) following the pipeline guide of the GATK (https://gatk.broadinstitute.org/hc/en-us/articles/360037593851-Mutect2) using the tumour-normal mode.

Then, all variants with a frequency > 0.001 in the gnomAD database (http://www.openbioinformatics.org/annovar/download/hg38_gnomad_genome.txt.gz) were removed using the filter functionalities of ANNOVAR, and all passed variants were annotated based on the refGene database (http://www.openbioinformatics.org/annovar/download/hg38_refGene.txt.gz).

Using the R package 'maftools' (v.2.8.0, https://github.com/PoisonAlien/maftools) for R (v.4.1.0; R Foundation for Statistical Computing, Vienna, Austria), an oncoplot was generated to display the top 20 somatic mutations in the samples. 568 mutated cancer driver genes were downloaded from https://www.intogen.org/search [37]. The method for mutational loads is described in the purity, ploidy, and somatic CN alteration section.

### Mutational signature

The R package 'deconstructSigs' (v.1.8.0, https://github.com/raerose01/deconstructSigs) was used to determine the linear combination of predefined signatures that most accurately reconstructed the mutational profile of a single tumour sample with the COSMIC v2 signature set [20].

### Purity, ploidy, and somatic CN alteration

The PURPLE toolkit (v.2.5.1) was downloaded from https://github.com/hartwigmedical/hmftools/releases/tag/purple-v2.51 to estimate the mutational load, purity, and CNAs. First, we used the paired normal/tumour mode of AMBER (v.3.2, https://github.com/hartwigmedical/hmftools/tree/master/amber#tumour-only mode) to generate a b-allele frequency file for use in PURPLE. Second, COBALT (v.1.1.0 https://github.com/hartwigmedical/hmftools/tree/master/cobalt#tumour-only-mode) was used to determine the read depth ratios of the tumour and normal reference genomes. The b-allele frequency and read depth ratio output from AMBER and COBALT, as well as somatic SNVs/indels from Mutect2, were passed to PURPLE. SV-input VCF files from gridss (v.2.9.4) were used to obtain the CN for the complex SV analysis pipeline. However, the SV input would oversegment the chromosome; therefore, this argument was ignored for the gain-time pipeline. The results of PURPLE included ploidy, purity, total CN, allele-specific CN, tumour mutational loads per megabase, and WGD status according to the major CN. WGD was considered true if more than 10 autosomes had a major allele CN > 1.5. A minor allele CN below one represents a LOH event.

Circos (v.0.69–6, https://github.com/vigsterkr/circos) was used to generate circularly composited renditions that showed somatic variants, tumour purity-adjusted CN changes, minor allele CNs, and SVs within or between the chromosomes.

### Subclonal composition and phylogenetic tree

Phyclowgs (https://github.com/morrislab/phylowgs) was used to integrate simple somatic mutations and CNA data to reconstruct tumour clones [38]. First, a phylowgs input parser (https://github.com/morrislab/phylowgs/tree/master/parser) was used to create simple somatic mutations from the Mutect2 output file and CN file from PURPLE. We limited the number of EBC7 variants used for phylogenetic reconstructions to 5000 to limit the run time. Subclone cancer cell fractions and phylogenetic trees were visualised using the R package ‘timescape’ (v.1.16.0, https://github.com/shahcompbio/timescape).

### Breakpoint and complex SV calling

Aligned bam files from the same patient were jointly used to call the breakpoints using gridss (v.2.9.4, https://github.com/PapenfussLab/gridss) [39], and this VCF file was passed to the R script 'gridss_somatic_filter' downloaded from https://github.com/PapenfussLab/gridss/blob/master/scripts/gridss_somatic_filter to remove variants with low confidence, or that could be found in a panel of normals. The R package 'fishhook' (v.0.1, https://github.com/mskilab/fishHook) was used to create contiguous tiles of 1 M and discover the enrichment regions of the breakpoint.

To discover various patterns of SVs, we used JaBbA (https://github.com/mskilab/JaBbA) to infer optimal CNs for DNA segments and bonds between segments to build a genome graph based on junctions and read depth. The first input file 'coverage' was generated from Cobalt, and the second input file 'JUNCTIONS' was generated from the gridss described above. The output of JaBbA was passed to the R package 'gnome' (v.0.1, https://github.com/mskilab/gGnome) to plot and classify subgraphs that satisfied the set of criteria corresponding to a particular class of simple (deletion [del], tandem duplication [dup], inversion [inv], inverted duplication [invdup], translocations [tra]) or complex (rigma, pyrgo, chromoplexy, chromothripsis, BFBs, DMs, and tyfonas) SVs [15].

### Timing of gain

We used the R package 'MutationTimeR' (v.1.00.2, https://github.com/gerstung-lab/MutationTimeR) to calculate CN-gain timing as described [27]. Four input files needed to be prepared: somatic mutations from the Mutect2 pipeline, CN file from PURPLE, complex SVs from the JaBbA-gGnome pipeline, and cluster file from phylowgs.

## Supplementary Information

**Additional file 1: Table1.** Characteristics, clinicopathologic features and outcomes of all patients.

**Additional file 2: Table 2. **Mutation loads for each sample.

**Additional file 3: Table 3.** SNV and indel.

**Additional file 4: Table 4.** mutational signature(COSMIC V2).

**Additional file 5: Table 5. **Allele gain and loss.

**Additional file 6: Table 6. **Phylowgs subclones cellular prevalence and CCF.

**Additional file 7: Table 7. **Her2 copy number.

**Additional file 8: Table 8. **Breakpoints enrichment tile.

**Additional file 9. **

**Additional file 10: Table 10. **Secondary affected genome and proportion.

**Additional file 11: Table 11. **Secondary gain time latency.

**Additional file 12: Table 12. **Chromosome arms that with large scale secondary gain and complex SV.

**Additional file 13: Supplemental Figure 1.** Overlap SNVs of primary tumor, lymph node metastasis, and liver metastasis of the 4 MBC patients. **Supplemental Figure 2.** The WGD status and complex SV of 5 metastatic breast cancer from a public database (http://mskilab.com/gGraph/).

## Data Availability

The information for analysis is in the Supplemental Tables. The raw sequence data reported in this paper have been deposited in the Genome Sequence Archive in National Genomics Data Center, China National Centre for bioinformation /Beijing Institute of Genomics, Chinese Acadamy of Science (HRA002540/PRJCA009821), which are publicly accessible at https://ngdc.cncb.ac.cn/gsa-human/s/zDT4ohn5.

## Abbreviations

BC: Breast cancer
BFBs: Breakage-fusion-bridge cycles
CN: Copy number
CNAs: Somatic copy number alterations
DMs: Double minutes
EBC: Early BC
FISH: Fluorescent in-situ hybridization
HER2: Human epidermal growth factor receptor 2
IHC: Immunohistochemistry
Indels: Insertions/deletions
LOH: Extensive loss of heterozygosity
MBC: Metastatic BC
SNVs: Single-nucleotide variants
SVs: Structural variations
WGD: Whole-genome doubling
WGS: Whole-genome sequencing
