# Supplementary material for: Characteristics of DNA macro-alterations in breast cancer with liver metastasis before treatment
Source: BMC Genomics. 2023 Jul 11;24:391. doi: 10.1186/s12864-023-09497-w (PMC10334641; doi:10.1186/s12864-023-09497-w)
Supplement: Supplementary file 13 — Additional file 13: Supplemental Figure 1. Overlap SNVs of primary tumor, lymph node metastasis, and liver metastasis of the 4 MBC patients. Supplemental Figure 2. The WGD status and complex SV of 5 metastatic breast cancer from a public database (http://mskilab.com/gGraph/). [file 12864_2023_9497_MOESM13_ESM.docx]

Supplemental Figure 1


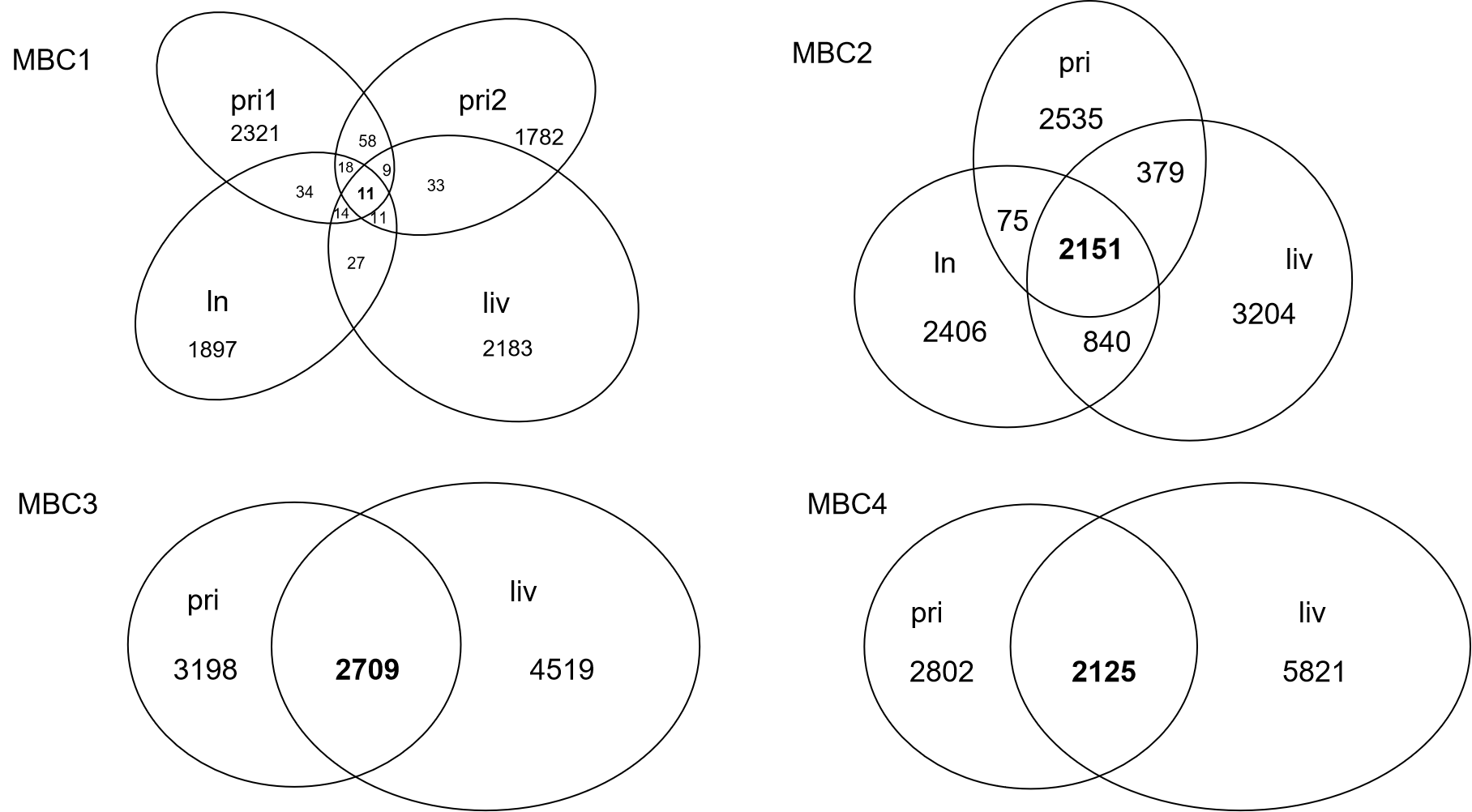


Overlap SNVs of primary tumor, lymph node metastasis, and liver metastasis of the 4 MBC patients.


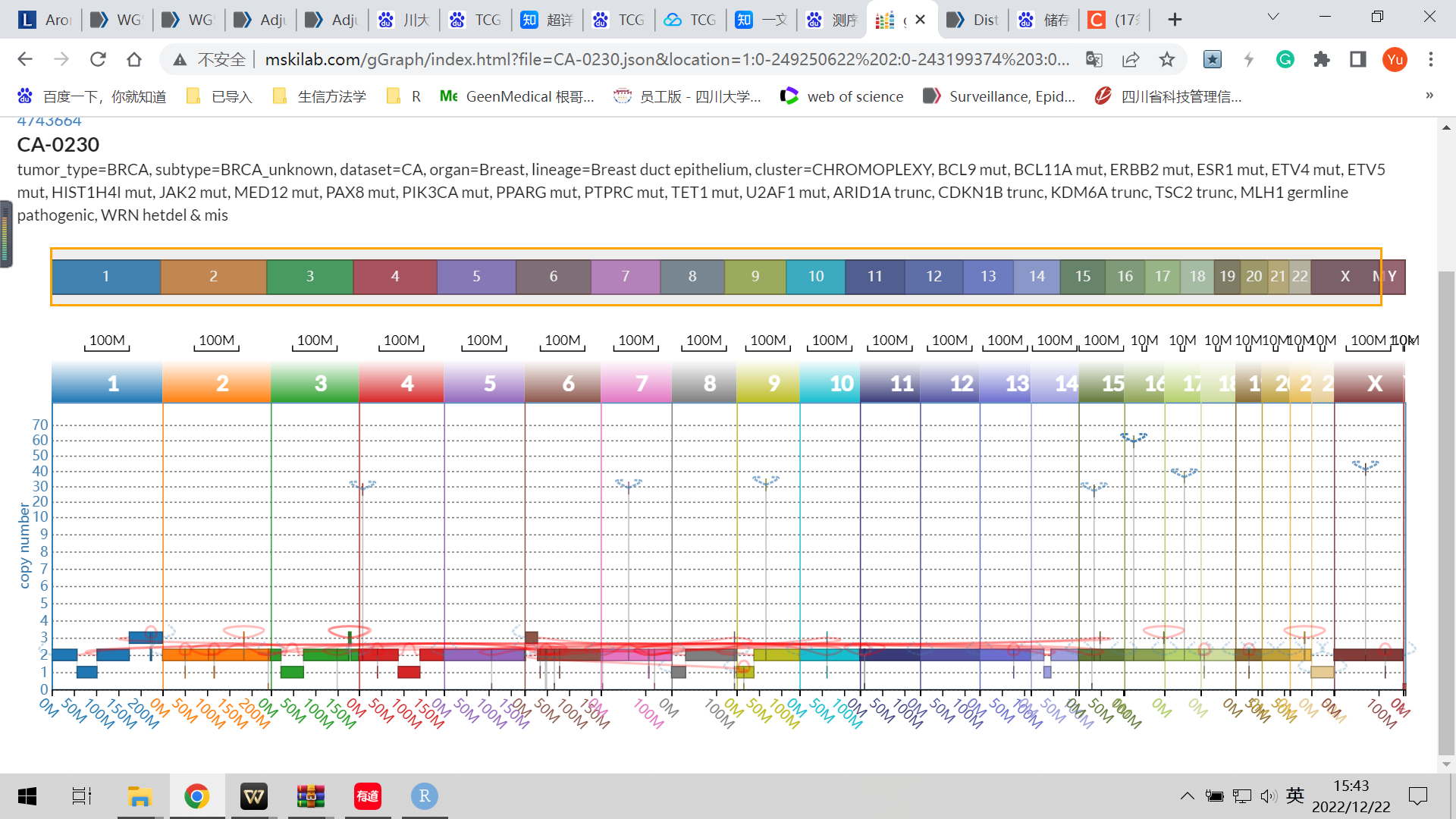

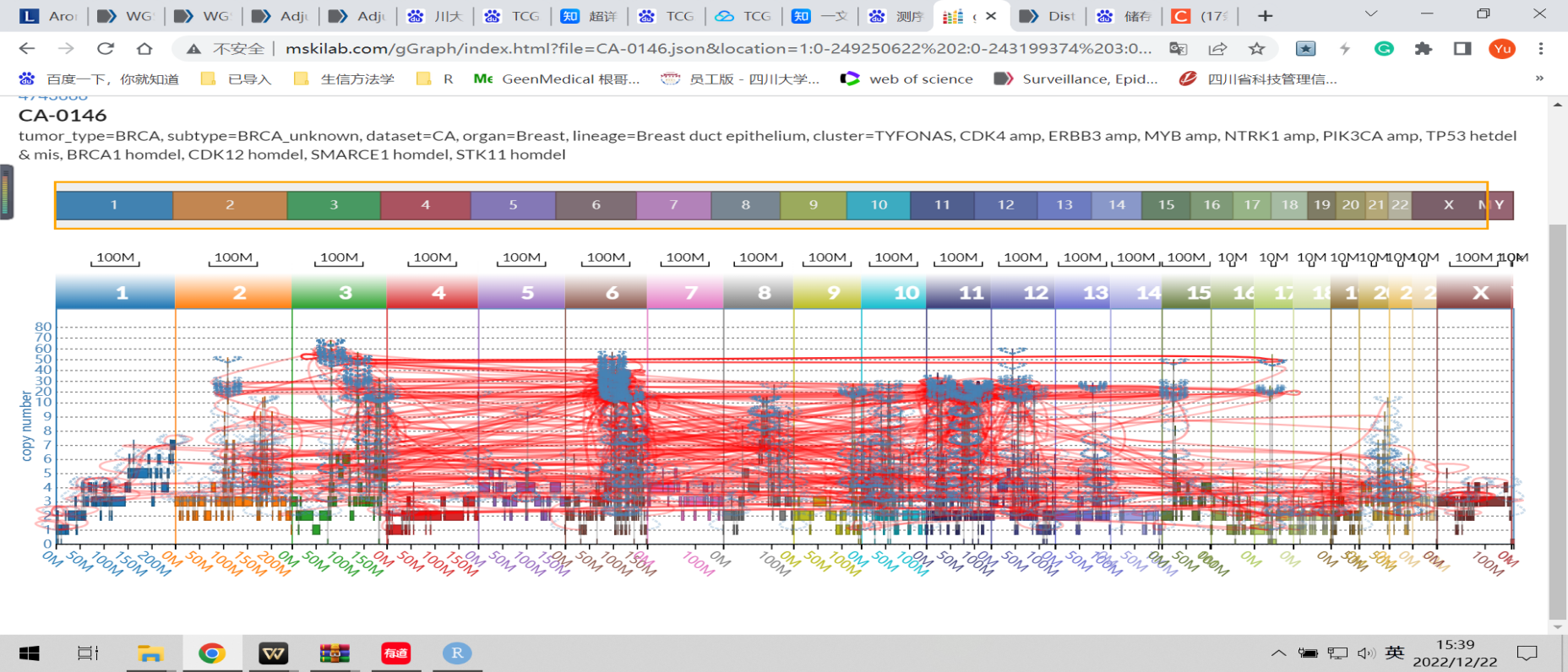

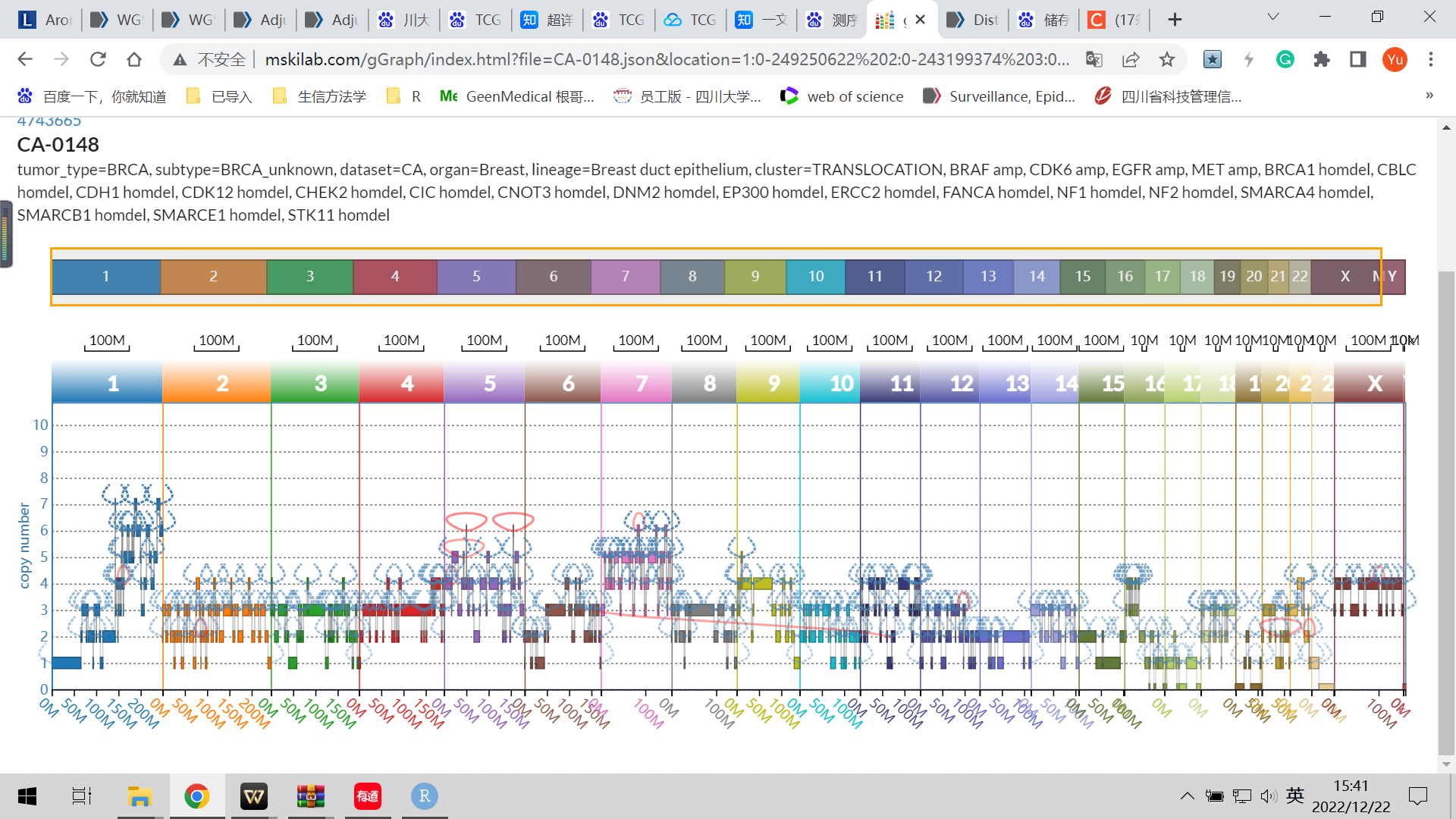

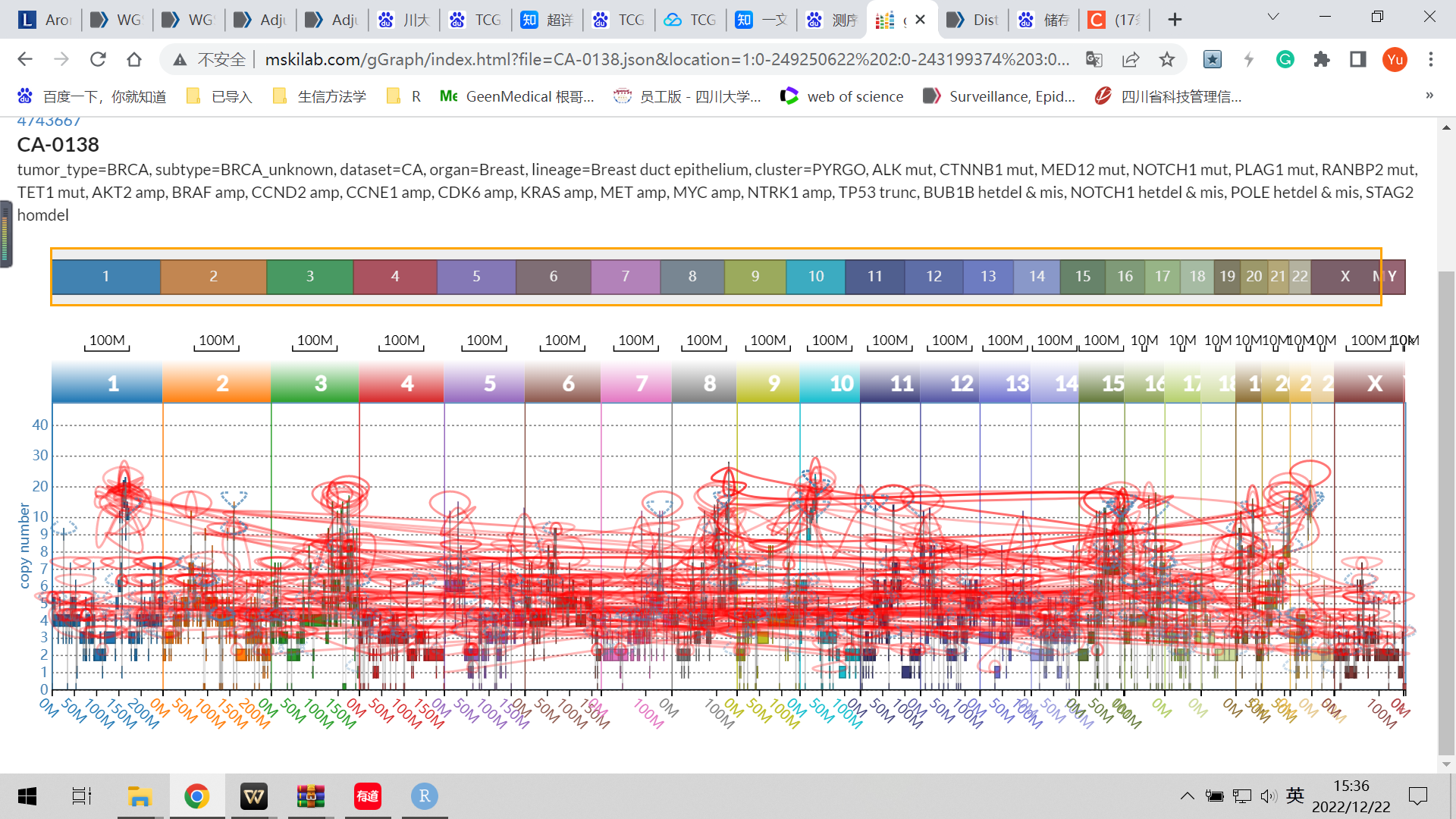

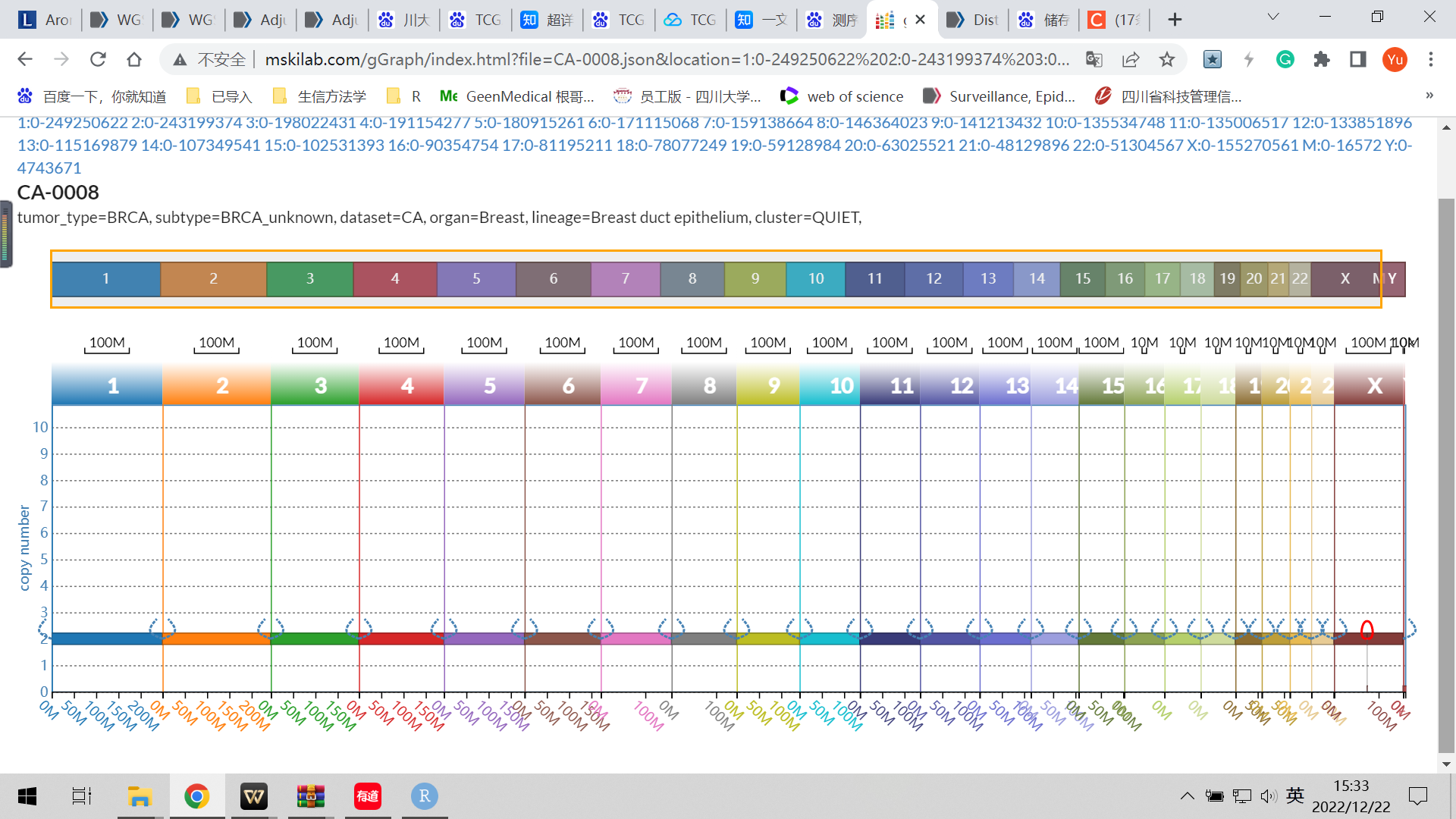
Supplemental Figure 2

The WGD status and complex SV of 5 metastatic breast cancer from a public database (http://mskilab.com/gGraph/)
